# Study Protocol: Understanding SARS-Cov-2 infection, immunity and its duration in care home residents and staff in England (VIVALDI)

**DOI:** 10.12688/wellcomeopenres.16193.2

**Published:** 2021-01-29

**Authors:** Maria Krutikov, Tom Palmer, Alasdair Donaldson, Fabiana Lorencatto, Gill Forbes, Andrew Copas, James Robson, Susan Hopkins, Paul Moss, Jeremy Farrar, Andrew Hayward, Laura Shallcross

**Affiliations:** 1 Institute of Health Informatics, University College London, London, NW1 2DA, UK; 2 Institute for Global Health, University College London, London, WC1N 1EH, UK; 3 Department of Health & Social Care, UK Government, London, SW1H 0EU, UK; 4 Centre for Behaviour Change, University College London, London, UK; 5 Four Seasons Health Care, Wilmslow, Cheshire, SK9 1BU, UK; 6 National Infection Service, Public Health England, London, NW9 5EQ, UK; 7 Institute of Immunology & Immunotherapy, University of Birmingham, Birmingham, UK; 8 The Wellcome Trust, London, UK; 9 Institute of Epidemiology & Health Care, University College London, London, WC1E 7HB, UK

**Keywords:** COVID-19, care homes, epidemiology, transmission, antibody, immunity, ageing, PCR

## Abstract

Global infection and mortality rates from severe acute respiratory syndrome coronavirus-2 (SARS-CoV-2) are disproportionately high in certain populations, including amongst older people. Care home residents are frequently exposed to infection due to contact with staff and other residents, and are highly susceptible to infection due to their age and co-morbidity. In England, official statistics suggest that at least 25% of all deaths in care home residents since the start of pandemic are linked to coronavirus disease 2019 (COVID-19), but limited testing for SARS-CoV-2 early in the pandemic means estimates of the true burden of disease are lacking. Additionally, little is known about patterns of transmission between care homes, the community and hospitals, or the relationship between infection and immunity in care home staff and residents. The VIVALDI study plans to address these questions.

VIVALDI is a prospective cohort study aiming to recruit  6,500 staff and 5000 residents from 105 care homes across England. Successive rounds of testing for infection will be performed over a period of 12 months.  Nasopharyngeal swabs will detect evidence of viral RNA and therefore active infection (accompanied by collection of data on symptoms), whereas blood tests will detect antibodies and evidence of cellular immunity to SARS-CoV-2. Whole genome sequencing of viral isolates to investigate pathways of transmission of infection is planned in collaboration with the COVID-19 Genomics UK Consortium. Qualitative interviews with care home staff will investigate the impact of the pandemic on ways of working and how test results influence infection control practices and behaviours. Data from residents and staff will be linked to national datasets on hospital admissions, antibody and PCR test results, mortality and care home characteristics.

Data generated will support national public health efforts to prevent transmission of COVID-19 and protect care home staff and residents from infection.

**Protocol registration**: ISRCTN14447421 05/06/2020

## Introduction

Severe acute respiratory syndrome coronavirus 2 (SARS-CoV-2) has caused a large number of deaths worldwide and rapidly changed how people live their lives by restricting social contact and daily activities. Early evidence pointed to the disproportionate impact of coronavirus disease 2019 (COVID-19) on older people, ethnic minorities and people with co-morbidities ^1, 2^. Care home residents have underlying risk factors for severe outcomes (age, comorbidity), but are also likely to have high rates of exposure to infection, through contact with care home staff or other residents. Studies from the USA, Canada and Europe have consistently shown high prevalence of SARS-CoV-2 in care home residents and staff, associated with significant mortality in residents ^3– 5^.

In England an estimated 450,000 individuals aged > 65 years live in approximately 9,000 care homes ^6^. Mortality data from the Office of National Statistics (ONS) suggests that >45,000 care home residents have died during the pandemic, although only 12,500 of these deaths were explicitly linked to COVID-19 ^7^. Accurate estimates of the burden of SARS-CoV-2 infection in care home residents and staff and the proportion of cases without symptoms are lacking because there has to date been limited testing for infection (antibody and RNA tests), and there is no comprehensive surveillance system for infection in care homes. We also have little insight into how infection transmits in the care home, both between staff and residents, and between care homes and other settings (community, hospitals), or how the pandemic has impacted on ways of working in the care home. The prevalence and duration of immunity to SARS-CoV-2 among staff and residents is also unknown.

The Department of Health & Social Care is currently rolling out infection (PCR) testing to all care home staff and residents ^8^. This will provide accurate data on the prevalence of infection across all care homes and insights into the types of care homes that are most likely to develop outbreaks. But this large-scale approach is not well-suited to assessing how outbreaks progress over time, or duration of immunity – information that is essential to inform the approach to testing in care homes for current and future pandemic waves. 

These questions can be answered most efficiently through a large prospective cohort study of care home staff and residents with repeat testing for infection and analysis of the immune response (antibody and cellular immunity). This will be combined with detailed data collection on symptoms and risk factors for infection, and linkage to NHS and public health data sources including viral sequencing. Through linkage to an existing study ( CATCH-19), we will also undertake qualitative interviews with 30 healthcare workers in a subset of care homes to gain insights into how the pandemic has impacted on healthcare staff and ways of working in the care home. This study is one of the largest undertaken in care homes, and will inform planning and the national public health response to COVID-19

This VIVALDI-2 study will be performed in conjunction with the VIVALDI-1 study. VIVALDI-1 is a cross-sectional survey of all 9000 care homes in England which provide dementia care or care to residents aged >65 years. The study is a collaboration between the ONS, Public Health England and UCL. The survey was delivered to care home managers and collected information on care home characteristics and staffing (number and type of staff, number of residents, sickness pay, use of agency staff), disease control measures (cohorting, isolation, care home closures) and cases of infection in staff and residents. Care home level responses will be linked to individual-level PCR results from the national surveillance whole care home testing data and analysed. Results from the survey are publicly available on the ONS website and have informed government policy on COVID-19 testing in care homes ^9^.

## Methods

### Study design

Prospective cohort study

### Settings

105 care homes in England run by the Four Seasons Health Care (FSHC) group.

### Participants

***Inclusion criteria.*** All residents aged > 65 years and all staff at participating FSHC care home sites; estimated as 11,500 potential participants (5,000 residents and 6,500 staff). Individuals who lack capacity to consent to study participation but have an available consultee can participate. Study materials will be translated into other languages, as necessary, to ensure inclusion of individuals who cannot speak English.

***Exclusion criteria.*** **None**.

### Study schedule

***Baseline assessments and blood sampling (antibody testing and cellular immunity).*** Demographic data and dates of care home entry and exit will be extracted from FSHC systems for all residents and healthcare staff. We will also extract information on care home characteristics (number of beds, residential and nursing care beds, number of patients with dementia, numbers of staff and type, staff turnover, care quality commission (CQC) rating, region, physical layout of the care home, number of visitors, number of suspected and confirmed SARS-CoV-2 cases, number of hospital admissions and deaths). 

Blood samples will be performed for baseline testing of antibody response and cellular immunity in all consenting staff and residents; and from residents who lack capacity provided there is strong justification for this (see section on consent).

***Intensive testing for infection (PCR, antibody testing and cellular immunity) and duration of the antibody and cellular immune response.*** All participants will undergo testing with nasopharyngeal swabs to detect SARS-CoV-2 viral RNA using PCR testing and blood testing to detect antibodies to this virus and cellular immunity. Two further rounds of PCR testing will be repeated at 7–28 day intervals with accompanying symptom data capture. Homes found to have active outbreaks will be prioritised for repeat testing earlier in this window.

Saliva will be collected alongside nasopharyngeal swabs in staff and residents in a subset of 4–8 care homes for PCR testing, to assess saliva as a diagnostic sample type.

Further rounds of blood sampling will be repeated at 6 weeks and 3 months in all participants and at 6 and 12 months in a subset of residents to assess the duration of the antibody response and whether cellular immunity wanes over time.

### Laboratory procedures

Laboratory tests will not be performed by the study team but will be contracted to external laboratories performing these tests as part of their current workload.

All PCR will be performed in national laboratories as part of the national testing programme on clinical isolates from nasopharyngeal swabs. Nasopharyngeal swabs will be carried out by trained care home staff or self-taken according to written instructions. These will be couriered on the same day to the central laboratory who will store them for testing.

PCR testing is performed using an Applied Biosytems 7500 Fast real time PCR system and using the Applied Biosystems TaqPath™ 1-Step Multiplex Master Mix (No ROX) (Cat. A28523) and TaqPath COVID-19-ASY-KIT 1000 (Cat. A47817). Primer sequence details are not currently published. Real time PCR cycling occurs as described in the Applied Biosystems guidelines and involves 1 cycle for 2 minutes at 25°C, 1 cycle for 10 minutes at 53°C, 1 cycle for 2 minutes at 95°C and then 40 cycles of 3 seconds at 95°C and 30 seconds at 60°C. A positive extraction control (Qnostics SARS-CoV-2 Positive Control cat. SCV2PC-075) and negative extraction control (RNAse-free water) is included in each plate. Quantification of the Positive/Negative/Inconclusive status of each sample is determined using the UgenTec FastFinder Interpretative software.

Blood samples will be collected by trained phlebotomists and will be couriered on the same day to the relevant laboratories for testing and storage.

Antibody tests will be performed using the Abbott *in vitro* SARS-CoV-2 IgG 6R86 assay using the ARCHITECT diagnostic platform which is widely used across NHS hospitals. These will be carried out in a commercial laboratory who will be contracted by the study team. 

Tests of cellular immunity will be performed on collected blood samples by the University of Birmingham study team using a separate protocol and ethics approval, which will be added to the data repository after it has been finalised.

### Data linkage

We will undertake quarterly data linkage to Hospital Episodes Statistics, Mortality data and virological test results from March 1 ^st^ 2020 onwards for all care home residents and staff. The legal basis for accessing identifiable data from all staff and residents without consent is provided through Covid-19: Notice under Regulation 3(4) of the Health Service Control of Patient Information Regulations 2002 (COPI) ^10^. Data linkage will continue for a period of up to 2 years as we anticipate that there will be additional waves of the pandemic in subsequent years.

### Whole genome sequencing

We will collaborate with the COVID-19 Genomics UK Consortium (COG-UK) to sequence viral samples derived from nasopharyngeal swabs from care home staff and residents and link results to epidemiological information (symptoms, dates of infection, admission to hospital, geographical location and care home layout). Sequencing data from care home residents and staff will be compared to sequences derived from hospitals and the community in the region surrounding each care home to make inferences about how SARS-CoV-2 spreads between care homes, the community and hospital settings. We will also explore patterns of transmission within the care home, and whether this varies dependent on whether the index case, as determined by mutations within the sequences and epidemiological data, was symptomatic or asymptomatic, or a member of staff or resident. We will also explore how care home factors such as building layout influence transmission of infection in the care home.

### Qualitative interviews with care home staff

We will undertake in-depth, semi-structured qualitative interviews with 30 care home staff from a sub-sample of participating care homes. This will be done through collaboration with an existing UCL-led study entitled *COVID-19 Impact and Burden in Care Homes (CATCH-19),* which is funded by the Economic and Social Research Council (ESRC) [ES/V003887/1] *.* FSHC will identify 6–8 potential care homes for inclusion in this sub-study, considering factors such as care home size, region and the number of COVID-19 cases reported by the care home.

We will use purposive, maximum variation sampling to recruit 3–5 participants per care home, from a range of staff roles (e.g. managers, care assistants, nurses). Interviews will be conducted in the months following the introduction of serological testing (months 2–5 of the study) and will explore the different ways in which the pandemic has impacted on care home staff, residents and ways of working, including: management of suspected or confirmed COVID-19 cases, interaction with other healthcare professionals and settings (e.g. primary and secondary care), access to medicines, advanced care planning, impact on non-COVID-19 care, and staff well-being. The interviews will also focus on how staff interpret and respond to PCR and antibody test results, as well as the subsequent impact on infection prevention control practices and staff behaviours. Specifically, the extent to which care home staff enact personal protective behaviours (i.e. use of PPE, social distancing in the work place) ^11^ and the barriers and enablers to these behaviours. The protocol and topic guide for the qualitative interviews have already been granted ethical approval by the UCL Research Ethics Committee (UCL REC ref: 13355/002) so details are not included in this protocol. 

### Data sources / measurement

The study will include primary data collection, extraction of information from electronic administrative data and linkage to existing medical records. Primary data collection will include nasopharyngeal swabs and saliva for PCR testing; blood samples for antibody testing and storage for analysis of the cellular immune response; data on symptoms of infection to accompany PCR testing; recording of ethnicity; information on care home characteristics and risk factors for infection such as the number of visitors; qualitative interview data.

Data will be extracted from care home records to find out the number of COVID-19 cases, hospital admissions and deaths by care home dating back to March 1 ^st^ 2020. Individual-level data will also be extracted for care home residents (age, gender, dates of entry and exit to and from the care home) and for care home staff (dates they started/stopped working at the care home). 

A brief symptom questionnaire will be performed at the time of nasopharyngeal sampling and for the subsequent 7 days to ascertain presence and development of symptoms. This will be performed by the care home manager. The questionnaire is available in the UCL Data Repository (extended data ^12^).

**Figure 1.**
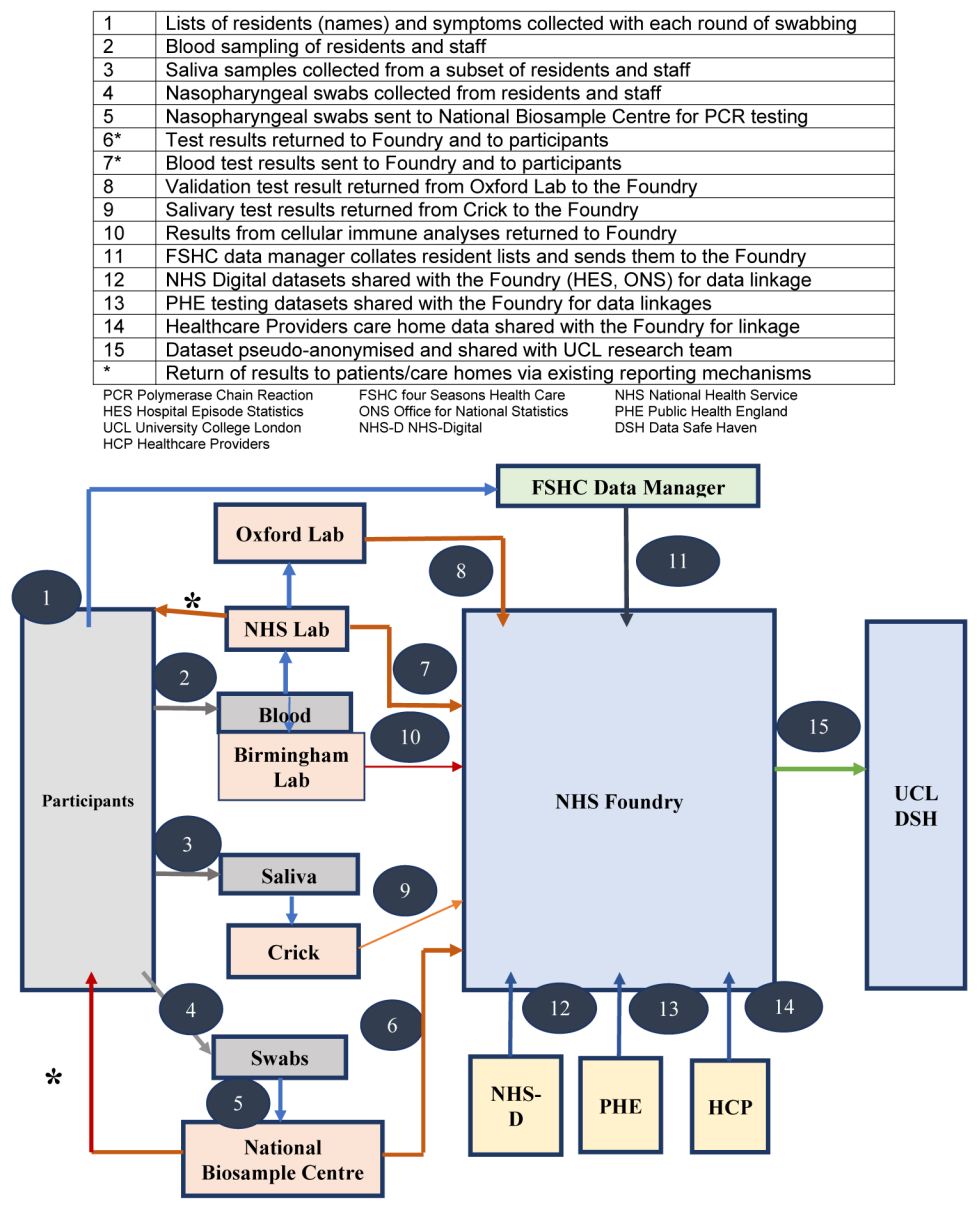
Data flows to enable safe, secure transfer of patient identifiable data and linkage.

Data linkage will be undertaken in the NHS Foundry and overseen and quality assured by NHS England. Figure 1 outlines the data flow for the study.

### Sample size

Assuming that around 80% of eligible staff and residents will participate, then on average approximately 38 residents and 49 staff will be tested per home across the 105 homes. Based on a conservatively estimated intra-cluster correlation of 0.36 (twice that reported for seasonal influenza ^13^) leads to effective sample sizes of around 279 residents and 285 staff, after accounting for the loss of precision due to clustering. Assuming an antibody prevalence of around 30% for both groups the precision of the estimate (based on 95% confidence interval width) will be ±5.4% for residents and for staff separately.

Importantly, as we anticipate that at least 1000 residents will have been infected at 3 months (using a conservative estimate of 20% infection prevalence), we should have sufficient precision and power to detect changes in antibody prevalence over time. For example, assuming 80% of infected residents agree to repeated antibody testing and 70% of these have an antibody response at 3 months (560 residents) then we can estimate the proportion of these retaining a response at 6 months within 4.2% assuming a retention rate of 50%. This assumes that antibody response is broadly unaffected by care home level factors.

### Outcomes

***Primary outcome***. The proportion of staff and residents who have previously been infected with SARS-CoV-2 based on presence of antibodies in serum at baseline, 6 weeks and 3 months.

***Secondary outcomes***

- The incidence and prevalence of acute infection and asymptomatic infection, at each round of swab testing
- The proportion of residents and staff with a new antibody response to SARS-CoV-2 at 3 months
- The proportion of residents with an antibody response to SARS-CoV-2 at 6 and 12 months, to investigate the duration of antibody response in this population
- The distribution of quantitative IgG and IgM responses to SARS-CoV-2 amongst staff and residents.
- Estimate the direct and indirect mortality in care home residents attributable to SARS-CoV-2
- Investigate transmission of SARS-CoV-2 between care homes, hospitals and the community by linking viral whole genome sequencing data from care home staff and residents to epidemiological information, and comparing sequences from the care home population to sequences that are circulating in local hospitals and the community
- Compare disease incidence, seroprevalence and symptom profiles, in care home residents and the general population
- Evaluate the performance of PCR to detect infection using saliva versus nasopharyngeal swabs
- Model the risk of importation of infection to care homes following discharge of a resident from hospital by linking to data on hospital admissions
- Use qualitative interviews to investigate the impact of the pandemic on ways of working in the care home, infection prevention control practices and behaviours, and to investigate how care home staff are interpreting and using Covid-19 test results.

### Data analysis and statistical analysis plan

A formal statistical analysis plan has been developed and can be accessed in the UCL data repository (extended data ^14^) and a summary is presented here. All data analyses will be performed using Stata version 15.

The sample proportions of staff and residents will be presented as summary statistics for the primary outcomes, together with the proportion of care homes with any antibody detection. Univariate logistic regression modelling with random effects for care home will identify risk factors for the primary outcome which will subsequently be included in a multiple regression model.

The rates of antibody acquisition, hospital admission and mortality will be presented. Duration of antibody response will be described by a Kaplan-Meier curve. Appropriate regression modelling will be performed to establish risk factors and associated variables with each outcome and will be presented as hazard, odds or rate ratios. Associations with loss of antibody response and risk of death will be investigated using Cox regression models. Poisson regression modelling will be conducted to investigate risk factors for new antibody development during study period and for hospital admissions.

All analyses will be conducted separately for staff and residents. The detailed statistical analysis plan will include a methodology to handle missing testing data.

The plan for analysis of viral sequencing data has not yet been developed. The topic guide and analysis plan for the qualitative interviews is described in the CATCH-19 protocol available in the UCL data repository (extended data ^15^).

### Consent

Informed consent to participate will be collected in writing from all care home staff. Wherever possible, written informed consent will be sought directly from residents. Study information materials will be translated into other languages upon request. 

For residents who lack capacity, the care home will contact a friend or next of kin to act as personal consultee. If a personal consultee cannot be identified, a nominated consultee, such as the care home manager or a member of staff will be asked to complete a professional declaration form. This approach meets the four criteria for HRA approval as set out in sections 31–33 of the Mental Capacity Act 2005 ^16^. This approach is also justified by the current lack of research in this population that can address important questions regarding impact and transmission of COVID-19.

### Data sharing

Research outputs will be published in open access journals and analytical code will be made available. A de-identified copy of the dataset will be made available for future research, however, decisions around the details shared will be made in partnership with FSHC. Fully anonymised transcripts from qualitative interviews will be archived in the UCL Research Data Repository, where they will be made accessible to other researchers.

### Ethics

The study has been approved by the South Central - Hampshire B Research Ethics Committee (reference number 20/SC/0238).

### Dissemination

Rapid results from this study will be shared with national advisory committees and organisations who are leading the pandemic response. Results will also be shared with Four Seasons Health Care and participants. Planned publication in a high impact peer-reviewed journal within one year of study end date.

### Study status

At the time of protocol submission, all participating sites had been identified and recruitment had started.

This study has been registered with ISCRTN under registration number 14447421 on the 5 ^th^ June 2020.

## Data availability

### Underlying data

No data are associated with this article.

### Extended data

UCL Data Repository: COVID-19 in care homes study (VIVALDI) - Symptom questionnaire.xlsx. https://doi.org/10.5522/04/12764441.v1 ^12^

- Symptom record V2 070720.xlsx (Symptom questionnaire administered to all study participants on the day of nasopharyngeal swab (SARS-CoV-2 PCR) that assesses for evidence of current infection)

UCL Data Repository: COVID-19 in care homes (VIVALDI) Statistical analysis plan. https://doi.org/10.5522/04/12821633.v1 ^14^

- VIVALDI analysis plan v1.2 final.pdf (Data analysis plan for VIVALDI-2 study performed by UCL researchers in 105 Four Seasons care homes across England)

UCL Data Repository: CATCH-19 topic guide. https://doi.org/10.5522/04/12821663.v1 ^15^

- CATCH-19_topic guide_V2_02.06.20.docx (Topic guide for CATCH-19 study qualitative interviews to be held with care home staff that are taking part in the VIVALDI study)

Data are available under the terms of the Creative Commons Attribution 4.0 International license (CC-BY 4.0).
