# Supplementary material for: Study Protocol: Understanding SARS-Cov-2 infection, immunity and its duration in care home residents and staff in England (VIVALDI)
Source: Wellcome Open Res. 2021 Jan 29;5:232. Originally published 2020 Oct 7. [Version 2] doi: 10.12688/wellcomeopenres.16193.2 (PMC7851710; doi:10.12688/wellcomeopenres.16193.2)
Supplement: Peer review history

# Peer review: Reviewer response for version 2

**DOI:** 10.21956/wellcomeopenres.18293.r42389

**Published:** 2021-02-01

**Authors:** Adam L Gordon

**Affiliations:** 1 Division of Rehabilitation & Ageing, University of Nottingham, Nottingham, UK

I am happy with this now.

Is the study design appropriate for the research question?

Yes

Is the rationale for, and objectives of, the study clearly described?

Yes

Are sufficient details of the methods provided to allow replication by others?

Yes

Are the datasets clearly presented in a useable and accessible format?

Yes

Reviewer Expertise:

NA

I confirm that I have read this submission and believe that I have an appropriate level of expertise to confirm that it is of an acceptable scientific standard.

# Peer review: Reviewer response for version 2

**DOI:** 10.21956/wellcomeopenres.18293.r42390

**Published:** 2021-02-01

**Authors:** Kanwar Hamza Shuja

**Affiliations:** 1 National Institute of Psychology (NIP), Quaid-I-Azam University, Islamabad, Pakistan; 2 Foundation University Rawalpindi Campus, Newlalazar, Rawalpindi Cantt, Pakistan

The authors have incorporated all the suggestions and the method section now seems more complete and elaborated.

Is the study design appropriate for the research question?

Yes

Is the rationale for, and objectives of, the study clearly described?

Yes

Are sufficient details of the methods provided to allow replication by others?

Yes

Are the datasets clearly presented in a useable and accessible format?

Not applicable

Reviewer Expertise:

Clinical psychology, criminal psychology, forensic psychology.

I confirm that I have read this submission and believe that I have an appropriate level of expertise to confirm that it is of an acceptable scientific standard.

# Peer review: Reviewer response for version 1

**DOI:** 10.21956/wellcomeopenres.17781.r41974

**Published:** 2021-01-19

**Authors:** Kanwar Hamza Shuja

**Affiliations:** 1 National Institute of Psychology (NIP), Quaid-I-Azam University, Islamabad, Pakistan; 2 Foundation University Rawalpindi Campus, Newlalazar, Rawalpindi Cantt, Pakistan

The article is a proposal of sort as it has proposed a plan for conducting a study using a cohort method. The introduction is written with a clear focus, explaining all the important points as it should, though, from the perspective of a layman, the authors should explain a little bit about what a VIVALDI cohort study is as it's not much even discussed in the method section. The rest of the method section though is written very scientifically and all the measures have clearly been explained. Since it is a longitudinal study there is no current data set present with this article as it is only proposing or laying out a plan for the study. This can be useful as the authors have written steps very detailed which can be used for replication purposes by other countries to assess the settings where COVID-19 is most prevalent. Overall the study in its current form has not added anything new but can be a useful article for the purpose of replication and in its follow-up studies depending upon how long the COVID-19 virus remains.

Is the study design appropriate for the research question?

Yes

Is the rationale for, and objectives of, the study clearly described?

Yes

Are sufficient details of the methods provided to allow replication by others?

Yes

Are the datasets clearly presented in a useable and accessible format?

Not applicable

Reviewer Expertise:

Clinical psychology, criminal psychology, forensic psychology.

I confirm that I have read this submission and believe that I have an appropriate level of expertise to confirm that it is of an acceptable scientific standard.

# Response

**Published:** 2021-01-26

**Authors:** Maria Krutikov

**Affiliations:** University College London, UK

We thank you for your review and evaluation of this manuscript.

# Peer review: Reviewer response for version 1

**DOI:** 10.21956/wellcomeopenres.17781.r41770

**Published:** 2021-01-18

**Authors:** Adam L Gordon

**Affiliations:** 1 Division of Rehabilitation & Ageing, University of Nottingham, Nottingham, UK

I found this a relatively straightforward and easy to follow protocol and I have only a couple of minor pointers.

 The authors use the world “elderly”, which PPI groups for older people consistently tell us is not acceptable.  The widely accepted term is “older people”.

 I was initially left confused about whether people who lack mental capacity to provide consent would be included.  I eventually decided that they must be, because there is a section at the bottom that talks about consultee procedures for residents who lack capacity.  Under inclusion and exclusion criteria the authors mention those who have capacity but don’t have the reading aptitude to engage with the study materials.  But they don’t make an explicit statement about those who lack capacity.  I would prefer to see them make a clear statement about inclusion of those who “lack capacity and have an available consultee”. As the authors have chosen to include care home staff as eligible consultees (ethics committees will have a variable opinion on this – but my guess is that they may have successfully used this approach during Vivaldi-I) then it’s likely that most people without capacity will be eligible for inclusion.  An explicit statement would be helpful.”

Is the study design appropriate for the research question?

Yes

Is the rationale for, and objectives of, the study clearly described?

Yes

Are sufficient details of the methods provided to allow replication by others?

Yes

Are the datasets clearly presented in a useable and accessible format?

Yes

Reviewer Expertise:

NA

I confirm that I have read this submission and believe that I have an appropriate level of expertise to confirm that it is of an acceptable scientific standard, however I have significant reservations, as outlined above.

# Response

**Published:** 2021-01-26

**Authors:** Maria Krutikov

**Affiliations:** University College London, UK

We thank you for your comments which we have addressed below:

1. Thank you for bringing our attention to the more widely accepted terminology.  We have changed the terms “elderly” to “older people” throughout the manuscript.
2. In order to clarify that we are including people with a consultee who lack the capacity to consent, we have added a statement that outlines this under the inclusion criteria section. We have also added in a clarification that people will not be excluded if they cannot speak English as we will translate study materials as necessary.
